# Psychological and Physical Health Improvements After Coronary Bypass: A Longitudinal Study in Cardiovascular Rehabilitation

**DOI:** 10.3390/ejihpe15100203

**Published:** 2025-10-07

**Authors:** Anna Panzeri, Giovanni Bruno, Giorgio Bertolotti, Andrea Spoto, Daniela Corbellini, Andrea Brandonisio, Ornella Bettinardi

**Affiliations:** 1 Department of General Psychology, University of Padua, Via Venezia 8, 35131 Padova, Italy; 2 IRCCS San Camillo Hospital, Via Alberoni, 70, 30126 Venezia, Italy; 3 Primary Care Psychology, Arona, 28041 Novara, Italy; 4 Casa di Cura Privata Accreditata San Giacomo, via San Bono 3, Ponte dell’Olio, 29028 Piacenza, Italyandrea.brandonisio@san-giacomo.it (A.B.); 5 Department of Mental Health AUSL of Piacenza, Via Anguissola, 15, 29121 Piacenza, Italy; o.bettinardi@ausl.pc.it

**Keywords:** anxiety, clinical psychology, coronary artery bypass graft, depression, health psychology, longitudinal study rehabilitation

## Abstract

**Background:** Patients who undergo coronary artery bypass graft (CABG) surgery often experience both physical and psychological challenges in the post-acute phase and thus follow an integrated rehabilitation program. **Objective:** This study aimed to examine changes in anxiety, depression, physical health, and mental health from admission to discharge and during a follow-up period up to 6 months after discharge. **Methods:** This study investigated longitudinal trends in the psychological and physical health of 608 patients (aged 65.75 ± 9.03 years, 80% male) undergoing a multidisciplinary rehabilitation program following CABG surgery. Repeated measures linear mixed models were used. **Results:** Significant reductions in depression (b = −7.30, *p* < 0.001) and anxiety (b = −2.22, *p* < 0.001) from admission to discharge were predicted by factors such as age (dep: b = 0.08, *p* < 0.001), male sex (dep: b = −1.15, *p* < 0.001), psychological symptoms (depression predicted by anxiety: b = 0.24, *p* < 0.001; anxiety predicted by depression: b = 1.25, *p* < 0.001), and the absence of preexisting stress (dep: b = 0.68, *p* < 0.001; anx: b = 1.68, *p* < 0.018). During the follow-up period from 45 days to 6 months postdischarge, physical health significantly improved (b = 3.77, *p* < 0.001), as predicted by age (b = −0.14, *p* < 0.001), male sex (b = 3.22, *p* < 0.001), mental health (b = 0.14, *p* < 0.001), and ejection fraction >35% (b = 3.56, *p* < 0.05). **Discussion:** These findings highlight the importance of considering both physical and psychological factors when designing rehabilitation programs for postacute CABG patients.

## 1. Introduction

Coronary artery disease is the most common heart disease in Europe and America and significantly contributes to mortality and disability in these regions (Virani et al., 2020). Individuals with severe angina and life-threatening ischemic heart disease may undergo coronary artery bypass graft (CABG) surgery, a well-established treatment designed to alleviate angina, decrease the risk of future heart attacks, extend life expectancy, and improve health-related quality of life (Alexander & Smith, 2016). Patients with cardiovascular disease may experience several psychological difficulties both before and after surgery (Rossi Ferrario et al., 2021), which can have consequences for their physical health and medical outcomes.

Research indicates that a substantial number of CABG patients experience psychological distress, including anxiety and depression. A study reported that over 80% of patients reported moderate-to-severe anxiety, with 31.8% exhibiting clinically relevant anxiety and 27.8% showing clinically significant depression postoperatively (Modica et al., 2018; Sawalha et al., 2024). Preoperative anxiety is common, affecting approximately 30% of patients, but it decreases significantly after surgery, dropping to 12% within one week and further to 6% at six months (Younes et al., 2019).

These emotional challenges can impede recovery and increase the risk of complications, as heightened anxiety is linked to increased pain perception and slower recovery rates (Sawalha et al., 2024; Younes et al., 2019). CABG patients face unique psychological challenges before and after surgery, which may significantly affect recovery outcomes; however, yet the precise dynamics of these factors remain underexplored (Modica et al., 2018; Sawalha et al., 2024).

Despite extensive research on the role of psychosocial factors in cardiovascular diseases (McKenzie et al., 2010), there is limited understanding of their longitudinal impact on psychophysical health, particularly in post-acute CABG patients (Albus et al., 2019b; Carola et al., 2024).

In this context, several studies have highlighted the fundamental role of psychosocial factors in the etiology, maintenance, and worsening of cardiovascular diseases. Notably, factors such as anxiety, depression, hostility and type A behavior, the psychosocial characteristics of work activity, and social isolation, affect the development and progression of cardiovascular diseases (Albus et al., 2019a; Blumenthal et al., 2003; Sommaruga et al., 2005; Task Force per le Attività di Psicologia in Cardiologia Riabilitativa e Preventiva, Gruppo Italiano di Cardiologia Riabilitativa e Preventiva, 2003).

These psychosocial factors can play both direct and indirect roles, promoting unhealthy lifestyles such as smoking, alcohol consumption, a sedentary lifestyle, and an unbalanced diet, which collectively facilitate disease relapse (Pietrabissa et al., 2017; Rozanski et al., 2023). Numerous studies have further confirmed the active role of psychosocial factors in the etiology and prognosis of cardiovascular diseases, demonstrating the possible role of depression, anxiety, chronic negative emotions, and related lifestyle factors (Chida & Steptoe, 2009; Kubzansky & Kawachi, 2000; Kuper, 2003; Ladwig et al., 2017).

Research has consistently demonstrated that psychological factors, such as depression and optimism, significantly influence the development and outcomes of cardiovascular diseases. Depression has been shown to negatively affect cardiovascular health, exacerbating disease progression and complicating recovery (Krittanawong et al., 2022). Conversely, optimism has been associated with a reduced risk of cardiovascular events and all-cause mortality, as highlighted by two systematic reviews (Krittanawong et al., 2022; Rozanski et al., 2023). These findings underscore the importance of addressing both negative and positive psychological factors in managing cardiovascular disease and improving long-term patient outcomes.

Medical guidelines recommend that participation in a structured rehabilitation program in the post-acute phase of CABG is necessary to complete treatment (Niebauer, 2016). Moreover, scientific evidence indicates that cardiac rehabilitation integrated with psychological intervention for patients following CABG can produce beneficial effects on anxiety and depression that are maintained over time (Jain et al., 2022; Pourafkari et al., 2016; Wells et al., 2021), helping patients cope better with changes in their health condition. In a Cochrane review by Richards et al. (2017), which included 35 randomized controlled trials with 10,703 participants (median follow-up: 12 months), compared with controls, psychological interventions significantly lowered depression symptoms (standardized mean difference [SMD] −0.27, 95%CI [−0.39–−0.15]), anxiety (SMD −0.24, 95%CI [−0.38–−0.09]), and stress (SMD −0.56, 95%CI [−0.88–−0.24]). Furthermore, patients receiving psychological interventions achieved a reduction in cardiovascular mortality (relative risk ratio: 0.79, 95%CI: 0.63–0.98).

Compared with exercise-based cardiac rehabilitation alone, additional psychological interventions aimed at lifestyle changes or distress management tended to reduce depressive symptoms; during a follow-up of five years, distress management was associated with a trend toward reduced cardiac morbidity (relative risk ratio: 0.74, 95% CI 0.51; 1.07) (Albus et al., 2019a). When this integration was achieved, an estimated 46% reduction in nonfatal cardiac events and a 41% reduction in mortality within two years were observed (Linden et al., 1996).

Therefore, within intensive multidisciplinary rehabilitation settings, identifying clinically significant levels of distress is crucial for recognizing patients who experience these issues and encouraging them to participate in educational and psychotherapeutic interventions integrated with conventional cardiac rehabilitation programs.

Höfer et al. (2005) strongly suggested that depression and anxiety symptoms significantly influence health-related quality of life (HRQL), suggesting that mediating factors such as depression and anxiety should be considered in clinical practice if HRQL is regarded as a clinical outcome.

### Research Gap and Objectives

To date, a gap has emerged in scientific literature. Despite the widely recognized importance of psychological factors, their effects on psychological and physical adaptation to illness remain unclear. Few studies have explored the impact of psychological factors on psychophysical health in post-acute patients undergoing bypass surgery, and even fewer have done so longitudinally. Recent findings concerning post-rehabilitation outcomes are controversial.

On the one hand, Pačarić et al. (2020), in their prospective study, reported that age, sex, lifestyle factors, and risk factors were not predictors of poor QoL assessments after rehabilitation. On the other hand, Tsai et al. (2019) reported that emotional symptoms—such as fatigue, sleep disturbances, depression, and anxiety—often persist for extended periods (Manzoni et al., 2018).

Considering this background, the present research aimed to investigate trends in psychological and physical health during and after completing a multidisciplinary rehabilitation program for patients who underwent CABG. Specifically, the study aimed to do the following:

- (a1) predict the trend of anxiety from admission to discharge on the basis of psychosocial factors (age, sex, stress, and depression)
- (a2) predicting the trend of depression from admission to discharge relying on psychosocial factors (age, sex, stress, and anxiety)
- (b1) predict the trend of physical health perceptions (measured with the SF-36) from 45 days post-discharge up to six months on the basis of psychosocial factors (age, sex, ejection fraction), including depression during rehabilitation.
- (b2) predict the trend of mental health perceptions (measured with the SF-36) from 45 days post-discharge up to six months, relying on psychosocial factors (age, sex, FE), including depression during rehabilitation.

Understanding the trends in psychological and physical health during rehabilitation could guide the development of tailored interventions to improve patient outcomes and reduce post-surgery complications. By investigating these factors longitudinally, this research seeks to fill a critical gap in understanding and provide actionable insights for improving the rehabilitation process for CABG patients.

## 2. Method

The study employed an observational longitudinal design to examine the longitudinal trends in psychological and physical health over time among patients in the post-acute phase following CABG surgery.

The inclusion criteria were being above 18 years of age or older, undergoing CABG, and being admitted to a rehabilitation program. The exclusion criteria included inability to complete assessments owing to cognitive or other deficits; lack of consent to use data for research purposes; and being under 18 years of age.

### 2.1. Procedure

Participants were enrolled in a three-week inpatient rehabilitation program at the ‘Casa di Cura San Giacomo’, Piacenza, Italy, from 1999 to 2007. The program involved rehabilitation activities conducted five consecutive days per week, following a diagnostic-evaluative and multidisciplinary treatment approach consistent with the Guidelines for Psychological Activities in Rehabilitative and Preventive Cardiology (Sommaruga et al., 2005, 2018; Task Force per le Attività di Psicologia in Cardiologia Riabilitativa e Preventiva, Gruppo Italiano di Cardiologia Riabilitativa e Preventiva, 2003), and was aligned with recommendations for comprehensive rehabilitation (Piepoli et al., 2010).

The sampling procedure consisted of consecutive recruitment of all eligible patients admitted to the rehabilitation program during the study period, on the basis of the inclusion and exclusion criteria described above. This consecutive sampling is a form of convenience sampling that aims to include all accessible patients meeting eligibility requirements, minimizing selection bias within the clinical setting. However, as this is not a probabilistic sampling method, the generalizability of the findings to broader populations may be limited.

#### Psychotherapeutic Care

As a part of the psychological program, all patients attended educational sessions to reduce behavioral risk factors and enhance subjective well-being (Albus et al., 2019a; Krittanawong et al., 2022; Sommaruga et al., 2018). When necessary, patients were offered individual sessions that included interviews and progressive muscle relaxation techniques following the abbreviated procedure of Bernstein and Borkovec (1973). Psychological intervention also aims to assess and enhance patients’ motivation to adhere to multidisciplinary rehabilitation programs, including compliance with diet and exercise (Pietrabissa et al., 2020). Patients who scored above the clinical cut-off on measures of anxiety or depression during psychological assessment were invited to additional individual sessions for brief psychotherapeutic support (Panzeri & Rossi Ferrario, 2020; Vedana et al., 2002).

### 2.2. Measures

The study utilized a range of medical and psychological assessment tools, including both hetero-administered and self-report questionnaires. All patients completed psychometric evaluations specifically designed for individuals undergoing intensive inpatient rehabilitation (Vedana et al., 2002).

Assessments were conducted at four time points:

- T0: at the start of the rehabilitation program;
- T1: at discharge from the rehabilitation program;
- T2: 45 days post-discharge;
- T3: Six months post-discharge.

At admission to the rehabilitation program, the following variables and constructs were recorded:

- demographic and social variables:
  - age, biological sex, education level, relational status, and occupation.
- medical variables:
  - information on the patient’s medical history and ongoing treatments.
  - The **New York Heart Association (NYHA) Classification** (J. A. Bennett et al., 2002): a tool for classifying the extent of heart failure, assigning patients to one of four categories on the basis of on their physical activity limitations and symptoms such as shortness of breath or angina pain. In this study, all 202 participants were classified as NYHA Class I, indicating no symptoms or limitations in ordinary physical activities (e.g., walking or climbing stairs).
  - **Ejection Fractional Percentage (EF%):** A measure of the heart’s efficiency in pumping blood, representing the percentage of oxygen-rich blood ejected from the left ventricle with each heartbeat. Normal EF values range between 50% and 70%, whereas values below 35% indicate cardiac dysfunction.

#### 2.2.1. Psychological Assessments During the Rehabilitation Period

At the beginning (T0) and end (T1) of the rehabilitation program, psychological assessments included the following:

- **Stress** was assessed by a physician at admission as a part of the risk factor analysis. Patients were categorized on the basis of whether they had experienced a period of intense stress recently (present =1, absent = 0)
- **Anxiety–Depression Questionnaire—in Rehabilitation (AD-R).** The AD-R Scale (Moroni et al., 2006) is a well-validated questionnaire, widely used in scientific research within the Italian context. The AD-R Scale, designed specifically for use with patients in rehabilitation, includes two key questionnaires. The first is an abbreviated form of the Depression Questionnaire (QD) (Vidotto et al., 2010). The QD-15 is a shortened version of the depression scale from the CBA, a well-established and validated assessment battery. The item reduction was conducted by the original authors and involved the removal of marginal items.
  Secondly, the A-scale is a version of the STAI-X3 for assessing anxiety. The STAI-X3 itself is a shortened version of the STAI-X1. The anxiety subscale, which consists of 10 items rated on a 4-point scale (ranging from 1 = “not at all” to 4 = “very much so”), yielding scores ranging from 10 to 40. The abbreviated Depression Questionnaire used in AD-R contains 15 items, referred to as QD-15.
  The depression subscale, which consists of 15 “Yes” or “No” items, with scores ranging 0–15. Gender-specific cutoffs indicate clinical symptoms: males scoring ≥22 and ≥6 signify state anxiety and depression symptoms, respectively, whereas females scoring ≥25 and ≥8 denote these conditions.
  The paper by Vedana et al. (2002) describes the development of the AD tool for rehabilitation purposes (State STAI and the QD scale from the CBA) (Bertolotti et al., 1990; Michielin et al., 2024; Sanavio et al., 2013).

#### 2.2.2. Follow-Up Measures

At 45 days (T2) and six months (T3) post-rehabilitation, the following measure was administered.

The Short-Form 36 of the Medical Outcomes Study (SF-36) (Apolone & Mosconi, 1998) is a widely used tool for assessing health related quality of life (HRQOL). The SF-36 includes 36 items grouped into the following eight subscales: physical functioning (PF), role limitation due to physical health (RP), bodily pain (BP), general health (GH), vitality (VT), social functioning (SF), role limitations due to emotional problems (RE), and mental health (MH).

These subscales contribute to two summary measures: the Physical Component Summary (PCS), which encompasses the subscales of physical functioning (PF), role limitations due to physical health issues (RP), bodily pain (BP), and general health perception (GH); and the Mental Component Summary (MCS), which gauges patients’ perspectives on vitality (VT), social functioning (SF), role limitations due to emotional issues (RE), and overall mental health (MH).

The Index of Life Quality (ILQ) provides a comprehensive measure of overall QoL by averaging PCS and MCS, thus proving a comprehensive index of HRQoL (Lins & Carvalho, 2016). Scores are obtained by summing item responses within a domain, dividing the total by the score range, and transforming the raw scores to a 0–100 scale. Higher scores indicate better functioning and overall health perceptions, making the SF-36 a key variable for assessing HRQOL.

### 2.3. Ethics Committee

The study protocol adhered to the standard care practices of ‘Casa di Cura San Giacomo’, Piacenza, Italy. No additional interventions were imposed on participants beyond standard treatment. The study was conducted in accordance with the principles outlined in the Declaration of Helsinki (World Medical Association, 2000). Ethics Committee approval was not required, as the activity falls within routine clinical care and the routine psychological support provided during rehabilitation, which was administered by licensed professionals (psychologists/psychotherapists) as part of the integrated care plan.

Ethical review and approval was obtained by the Scientific and Medical Director of the Institute, who oversees the research activities within the Institution to ensure compliance with ethical standards and clinical governance.

Before participation, all participants voluntarily provided informed consent for the use of their anonymized clinical data for research purposes. Personal identifiers were removed to maintain confidentiality and protect participants’ privacy throughout the study.

### 2.4. Statistical Analysis

All the statistical analyses were performed using R software in the RStudio environment (version 4.3.3) (R Core Team, 2023; RStudio Team, 2023). The primary analysis utilized the *lme4* package for fitting linear mixed-effects models (Bates et al., 2023). Although some missing data were present in the dataset, only complete cases were included in the analyses. This approach was chosen to avoid the introduction of bias or uncertainty associated with imputation methods, and to maintain a straightforward analytical process (Little & Rubin, 2019; Sterne et al., 2009).

Regarding model specification, four linear mixed-effects models with repeated measures were constructed to examine longitudinal changes in the following outcome variables: anxiety, depression, physical health, and mental health. For each model: the fixed effects included time (treated as a categorical variable), sex (males vs. females), age as a continuous variable, and the presence (=1) or absence (=0) of stress hetero-evaluated by the clinician. Random intercepts were included for each patient (grouping variable: v1cod) to account for within-subject correlation due to repeated measurements.

The following parameters were estimated and reported in each model. The residual variance (σ^2^) represents the individual-level variability, which is unexplained after accounting for both fixed and random effects, it quantifies how much the outcome variable varies within each participant over time. Intercept variance for the grouping variable (τ_00 v1cod_) is the variance of the random intercepts for individuals (_v1cod_) and reflects the variability in baseline levels of the outcome across different participants, representing between-individual variance.

The Intraclass Correlation Coefficient (ICC) indicates the proportion of total variance in the outcome that is attributable to differences between groups (participants). It is calculated as the ratio of the between-group variance (τ_00_) to the total variance (τ_00_ + σ^2^). Higher ICC values suggest that a larger proportion of the total variance is due to differences between individuals (i.e., greater clustering or similarity within individuals over time). The Number of Groups (N_v1cod_) refers to the total number of participants.

To ensure model validity, the assumptions (e.g., normality of residuals, homoscedasticity, absence of multicollinearity) were tested and were not violated. To evaluate model fit, both marginal and conditional R^2^ values for the linear mixed-effects models were reported. Marginal R^2^ represents the proportion of variance explained by the fixed effects alone, reflecting the contribution of the predictors common to all participants. The conditional R^2^ represents the proportion of variance explained by the combination of fixed and random effects, capturing the total variance explained by the entire model including individual-specific variability. The conditional R^2^ indicates how much additional variance is accounted for by the random effects (e.g., differences between participants).

With respect to the significance testing, all the statistical tests were two-sided, and a *p*-value of <0.05 was considered statistically significant. For each fixed effect, we reported the estimated coefficients, standard errors, 95% confidence intervals (95%CIs), and *p*-values.

## 3. Results

### 3.1. Participants

The sample consisted of 608 consecutively enrolled patients who had undergone CABG, and in the post-acute phase, were admitted to a subsequent multidisciplinary rehabilitation program at ‘Casa di Cura San Giacomo’, Piacenza, Italy.

Their ages ranged from 37 to 85 years, with an age of 65.75 ± 9.03 years.

Regarding biological sex, most participants were male (80%), whereas 20% were female. Regarding education, most participants completed elementary school (46.36%), followed by middle school (24.18%), high school (18.36%), a bachelor’s degree (4.91%), or no formal education (6.18%).

Concerning the current occupation of the patients in this sample, the majority were housekeepers (71.55%), followed by employees (5.92%), managers (4.93%), workers (4.27%), retirees (3.29%), unemployed individuals (0.99%), entrepreneurs/freelancers (0.82%), craftsmen/tradesmen (0.33%), and others (5.75%).

Most patients were classified as NYHA class 1, indicating no limitations (94.1%) and good cardiac efficiency with an EF >0.35% (95.5%). Additionally, 69.6% did not experience post-surgery complications. Among the total sample, 382 patients underwent psychological treatment (56.18%), with the number of interventions ranging from 1 to 6.

Table 1 reports the descriptive statistics of the sample.

### 3.2. Repeated Measures Linear Mixed Models

Table 2 shows that from admission to discharge, depression scores significantly decreased (b = −7.30, se = 1.13, *p* < 0.001). This reduction was predicted by sex (b = −1.15, se = 0.32, *p* < 0.001), age (b = 0.08, se = 0.01, *p* < 0.001), anxiety (b = 0.24, se = 0.01, *p* < 0.001), and the presence of preexistent stress (b = 0.68, se = 0.33, *p* < 0.001). The model’s conditional R^2^ was 0.716.

**Figure 1:**
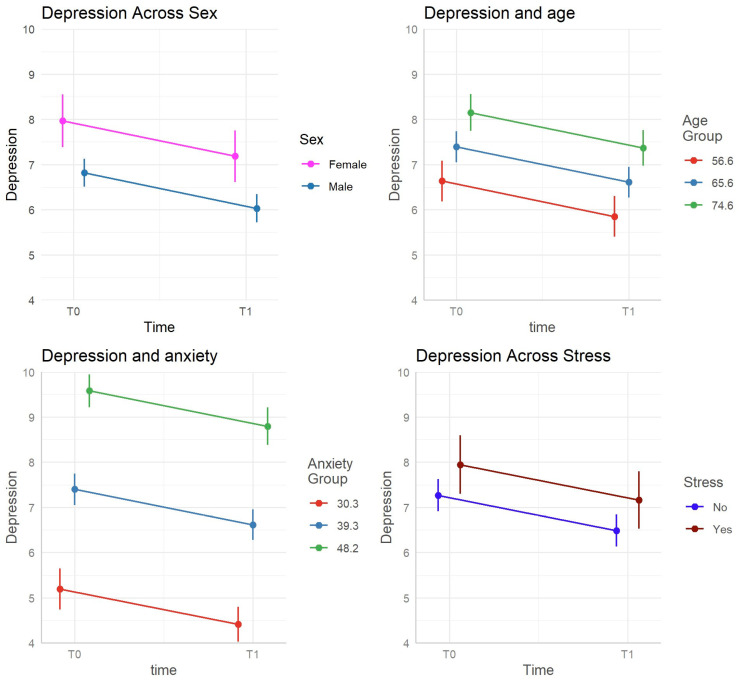
Graphical representation of the effects of the model on depression.

Figure 1 shows the effect of the model on depression. From admission to discharge, anxiety scores showed a statistically significant reduction (b = −2.22, se = 0.36, *p* < 0.001). This reduction was predicted by depression (b = 1.25, se = 0.06, *p* < 0.001) and the presence of preexistent stress (b = 1.68, se = 0.71, *p* < 0.018). The model’s conditional R^2^ was 0.670.

Table 3 shows that during the post-discharge period, from 45 days to 6 months after discharge, the general physical health score (PCS measured with the SF-36) showed a statistically significant improvement (b = 3.77, se = 0.54, *p* < 0.001).

This improvement was predicted by male biological sex (b = 3.22, se = 0.82, *p* < 0.001), age (b = −0.14, se = 0.04, *p* < 0.001), the trend of depression from admission to discharge (b = −0.29, se = 0.08, *p* < 0.001), the general mental health scores measured with the MCS of SF-36 (b = 0.14, se = 0.03, *p* < 0.001), and EF > 35% (b = 3.56, SE = 1.50, *p* = 0.018). The model’s conditional R^2^ was 0.535 showing that more than half of variance of the dependent variable was explained.

During the post-discharge period, from 45 days to 6 months after discharge, the general mental health score (MCS measured with the SF-36) did not show any statistically significant change (b = −1.06, se = 0.62, *p* = 0.088). Mental health was predicted by age (b = 0.11, se = 0.04, *p* < 0.001), the trend of depression from admission to discharge (b = −0.81, se = 0.09, *p* < 0.001), and the general physical health score (b = 0.18, se = 0.04, *p* < 0.001). The model’s conditional R^2^ was 0.535.

The mixed-effects model examining the ILQ derived from the SF-36 revealed a significant improvement over time, with ILQ scores increasing by an average of 3.91 points from time T2 to T3 (b = 3.91, *p* < 0.001) and those with EFs greater than 35% showed significantly higher ILQ scores (b = 6.81, *p* = 0.035), compared to those with EF<35%.

Higher levels of anxiety and depression were both significantly associated with lower ILQ scores (anxiety: b = −0.31, *p* < 0.001; depression: b = −0.88, *p* < 0.001).

Sex showed a non-statistically significant trend toward higher ILQ in males than in females (b = 3.16, *p* = 0.067), whereas age and stress were not statistically significant predictors.

## 4. Discussion

The present observational research study aimed to explore the longitudinal trends in psychological and physical health among patients who underwent CABG surgery, from their admission to an integrated rehabilitation program up to six months post-discharge. These findings suggest potential associations between psychological variables and post-rehabilitation outcomes; however, due to the observational design and lack of a control group, causal interpretations cannot be made, and the results should be viewed as exploratory and preliminary.

Findings from the mixed models revealed significant longitudinal patterns, both from admission to discharge and during the follow-up period. From admission to discharge, both depression and anxiety scores significantly decreased. Depression was positively predicted by anxiety, sex (with females showing significantly higher depression levels than males), age (with older age associated with greater depression), and the presence of stress before surgery, which exposed patients to elevated depression levels. The model explained a substantial amount of variance in depression (R^2^ = 0.716), underscoring its robustness. Similarly, anxiety levels were significantly predicted by higher depression levels, and the presence of preexistent stress, with a good amount of variance in anxiety explained by the model (R^2^ = 0.670).

Considering the post-discharge follow-up, physical health showed a statistically significant and sustained improvement from 45 days after discharge up to 6 months (R^2^ = 0.535), indicating that more than half of the variance in physical health outcomes was explained by the model. Several factors were significant predictors of better physical health during this recovery phase. Being male was associated with more favorable physical health outcomes, which may reflect sex-related differences in recovery trajectories or physiological resilience (K. M. Bennett et al., 2023). Younger age was also associated with better physical health, which is consistent with the general understanding that younger individuals tend to have greater regenerative capacity and fewer comorbidities that could impede recovery. Better mental health was positively associated, highlighting the important interplay between psychological well-being and physical recovery, possibly through mechanisms such as motivation, adherence to rehabilitation, and reduced stress-related physiological burden. An EF greater than 35% was linked to improved physical health, underscoring the critical role of preserved cardiac function in facilitating recovery and physical performance post-discharge.

Conversely, mental health values remained stable and did significantly change from 45 days to 6 months post-discharge. However, levels of mental health were positively associated with better physical health, the absence of stress, and greater age—which is consistent with scientific evidence suggesting that greater age is associated with better emotion regulation strategies (Kaufman et al., 2016; Orgeta, 2009).

This stability of mental health during follow-up may reflect several factors. First, the mental health measure used captures a broad range of psychological domains, potentially diluting sensitivity to changes in specific clinical symptoms (e.g., anxiety, depression). As a follow-up outcome assessment, it is recommended to include not only a QoL evaluation but also more targeted measures of anxiety and depression that address the primary clinical conditions of individuals. Second, it is possible that mental health improvements occur earlier during hospitalization or immediately post-discharge, with a plateau phase during longer-term recovery. Third, stability might indicate resilience or effective coping mechanisms in the sample, especially considering that greater age was associated with better mental health, which is consistent with the literature suggesting improved emotion regulation with age (Contreras et al., 2024; Kaufman et al., 2016; Orgeta, 2009). Furthermore, the lack of significant change could be influenced by ceiling effects, limited variability in mental health scores at follow-up, or external factors (e.g., social support or private rehabilitation programs) not captured in this study. Importantly, mental health trajectories may vary individually, and group-level analyses might mask meaningful changes in subgroups. Given these considerations, future studies should incorporate more targeted and sensitive measures of specific psychological constructs such as anxiety and depression during follow-up, alongside broader QoL assessments. Additionally, longer follow-up periods and the inclusion of potential moderating variables (e.g., social support, coping strategies) may better elucidate the dynamics of mental health recovery after discharge (Helgeson et al., 2018; Panzeri et al., 2024).

Additionally, these findings showed that ILQ improved significantly over time post-discharge (T3 vs. T2). Better cardiac function (EF > 35%) was associated with higher ILQ, whereas higher anxiety and depression scores were linked to lower ILQ. Sex tended to favor males, but age and stress were not significant predictors in this model.

Overall, these findings highlight that patients who participated in a multidisciplinary rehabilitation program during the post-acute phase following CABG surgery experienced significant improvements in mental and physical health, with notable progress in depression symptoms. Furthermore, these findings identify factors that are associated with the psychophysical outcomes of patients undergoing CABG, offering valuable insights for clinical practice and suggesting further investigations of their role as protective or risk factors.

These findings align with evidence from the scientific literature in the field and nicely add to the extant literature. Ma et al. (2020) reported similar results in a comprehensive rehabilitation and intensive education (CRIE) program for patients with unprotected left main coronary artery disease who underwent CABG, demonstrating its efficacy in improving anxiety, depression, and QoL, and reducing major adverse cardiac and cerebrovascular events. Furthermore, Geulayov et al. (2018) reported that depression symptoms one year post-surgery were positively associated with mortality, with limited evidence for sex differences. This underscores the importance of ongoing assessment for depressive symptoms in individuals with clinical depression scores who may benefit from psychological support. Additionally, these findings resonate with the scientific literature identifying preexisting psychological stress—potentially stemming from traumatic experiences—as significant risk factor for both the mental and physical health of patients following rehabilitation for cardiovascular diseases, including CABG (Rossi et al., 2022; Rossi et al., 2024a; Rossi et al., 2024b; Rossi et al., 2024c; Vilchinsky et al., 2017).

### 4.1. Limitations

Some limitations of this research need to be acknowledged. The study design has some limitations due to its observational nature. First, the absence of a control group limits the study internal validity and prevents causal interpretation of the effects of the rehabilitation program, necessitating cautious interpretation of the results. Future research should consider alternative approaches (e.g., historical controls, subgroup analyses) to strengthen causal inference. Second, the study employed a single-center, consecutive sampling design, which inherently restricts the broader applicability of the findings. Because all participants were recruited from one healthcare facility, the sample is likely to reflect the specific demographic, clinical characteristics, and care practices unique to that center. As a result, the findings may not be fully representative of the wider population of post-CABG patients in different geographic regions, healthcare systems, or varying clinical environments. This limitation raises concerns about external validity, as the homogeneity of the sample could reduce the ability to generalize results to more diverse patient groups. Additionally, consecutive sampling, while convenient and practical, may introduce selection bias if certain patient subgroups are underrepresented or if referral patterns and inclusion criteria vary over time. Together, these factors may influence the observed outcomes and limit the extent to which the results can be extrapolated beyond the specific context of this study.

Patients who engage in rehabilitation programs might differ systematically from those who do not (e.g., motivation, access to care), which could influence outcomes. A third limitation is the lack of external validation of these models, which limits the generalizability and robustness of the findings to other populations. Furthermore, potential sample selection bias stemming from the recruitment methods or inclusion criteria may affect the representativeness of our findings and their broader applicability. Moreover, in line with the research hypotheses, a selection of variables was considered and entered into the models. However, other unmeasured variables or factors, such as social support, which be crucial for hospitalized patients may also play a role (Compare et al., 2013; Panzeri et al., 2022; Ratti et al., 2017). Importantly, a measure of state anxiety (i.e., “at this moment”) was used, whereas a measure of general anxiety symptoms might have provided a more comprehensive depiction of the patients’ psychological condition during this period. Although the AD-R instrument has been validated within the Italian rehabilitation context, it is less widely utilized internationally; therefore, future research could benefit from employing more globally standardized measures. Cases with missing data were excluded but this procedure may introduce selection bias, which means that the results should be carefully interpreted (Little & Rubin, 2019; Sterne et al., 2009).

### 4.2. Strengths

The present research study has several strengths regarding its novel contribution. First, it was conducted with a large clinical sample of post-acute inpatients who underwent CABG and were admitted to a multidisciplinary rehabilitation program. This study presents novel findings based on data that are unique to this analysis and have not been previously published or analyzed in any prior reports; thus, they represent new contributions to the literature. Second, another important strength is the longitudinal design, which followed patients from admission to rehabilitation, through discharge, and up to six months post-discharge, enabling the monitoring of their psychophysical health over time. Third, the assessment integrated hetero-administered interviews with physicians and self-report questionnaires to measure psychological constructs, all of which are well-validated and standardized. The large sample size is representative of the CABG patient population and allows for the generalization of these findings. Moreover, the large sample size enabled precise and accurate estimates through repeated measures mixed models, a robust and advanced statistical analysis technique (Brown, 2021; Meteyard & Davies, 2020; Panzeri et al., 2023).

### 4.3. Future Research

Future research could adopt a more robust study design by including a comparison group, as in randomized controlled trials, to specifically test the effects of psychological interventions. Future studies might also consider different ethnic groups, explore gender differences, control for other potential variables, and examine the relationships among various factors. For example, certain factors that may influence the process of adaptation to cardiovascular disease, such as levels of acceptance and forgiveness, both of oneself and of the illness, remain underexplored in the literature (Consoli et al., 2020; Hughes et al., 2018; Rossi et al., 2022). Future research on the psychological effects of CABG surgery should focus on investigating targeted interventions to reduce anxiety and depression such as cognitive-behavioral therapy and mindfulness-based approaches (Dao et al., 2011; Freedland et al., 2009), as well as exploring the moderating role of transdiagnostic cognitive factors such as intolerance of uncertainty in managing illness-related uncertainty in every day life (Bottesi et al., 2017, 2019; Zhang, 2017). Additionally, exploring collaborative care models that integrate mental health support within cardiac rehabilitation programs could enhance overall patient outcomes. Longitudinal studies are needed to track psychological symptoms over time, identify critical periods for intervention, and examine the impact of preoperative psychological assessments on postoperative outcomes (Oxlad & Wade, 2008; Spezzaferri et al., 2009). Finally, comparative effectiveness research should evaluate different rehabilitation models to determine which best supports mental health recovery in CABG patients (Subih et al., 2024).

## 5. Conclusions

In conclusion, the results of this longitudinal study highlight factors associated with the psycho-physical health of patients after CABG, suggesting important implications for both research and clinical practice. A recent review by Richards et al. (2017) revealed that psychological interventions for coronary heart disease led to reductions in cardiac mortality rates and alleviated psychological symptoms (e.g., depression, anxiety, and stress). However, significant uncertainty remains regarding which individuals would benefit most from these interventions (e.g., those with or without psychological disorders at baseline) and the specific components of successful interventions. Based on our experience, a brief depression assessment could be useful for identifying individuals who maintain clinically significant levels of depression symptoms at the end of the rehabilitation period.

### Relevance to Clinical Practice

The present research provides valuable evidence to inform clinical professionals about the protective and risk factors affecting the psychophysical health of patients who have undergone CABG surgery and rehabilitation. Particular attention should be given to older patients, those with preexistent stress, those with co-occurring anxiety or depression, and females. These findings emphasize the strong interconnection between physical and mental health, which mutually influence one another over time. This is especially significant for multidisciplinary rehabilitation programs and for health psychology in medical settings.

## Figures and Tables

**Table 1:** Descriptive statistics of the variables measured in the sample.

	Females (*n* = 121, 20%)	Males (*n* = 487, 80%)	Total (*N* = 608)
Age, mean (SD)	69.76 (8.12)	64.76 (8.97)	65.75 (9.03)
NYHA Class 1—no limitations	90 (93.8%)	402 (94.1%)	492 (94.1%)
NYHA Class 2—mild limitations	6 (6.2%)	24 (5.6%)	30 (5.7%)
NYHA Class 3—significant limitations	0 (0.0%)	1 (0.2%)	1 (0.2%)
Ejection Fraction < 35% (impaired)	5 (4.5%)	21 (4.5%)	26 (4.5%)
Ejection Fraction > 35%	107 (95.5%)	443 (95.5%)	550 (95.5%)
Complications post-surgery	30 (27.0%)	144 (31.2%)	174 (30.4%)
Anxiety (T0), mean (SD)	45.44 (9.77)	40.90 (9.08)	41.80 (9.34)
Depression (T0), mean (SD)	9.74 (4.40)	7.24 (4.05)	7.74 (4.24)
Anxiety (T1), mean (SD)	40.17 (8.13)	36.37 (7.79)	37.13 (8.00)
Depression (T1), mean (SD)	7.95 (3.95)	5.22 (3.92)	5.76 (4.07)
PCS at 45 days, mean (SD)	37.99 (7.33)	42.95 (7.93)	42.10 (8.04)
Range	24.50–54.54	21.80–69.13	21.80–69.13
MCS at 45 days, mean (SD)	43.84 (11.28)	47.35 (10.29)	46.75 (10.52)
Range	26.88–65.42	14.36–68.13	14.36–68.13
Index of Life Quality (ILQ) at 45 days, mean (SD)	40.92 (7.04)	45.15 (7.13)	44.43 (7.28)
Range	29.28–54.73	25.28–59.83	25.28–59.83
PCS at 6 months, mean (SD)	42.10 (8.85)	47.32 (8.61)	46.28 (8.90)
Range	20.46–61.57	21.24–67.28	20.46–67.28
MCS 6 months, mean (SD)	46.62 (11.52)	48.93 (9.75)	48.47 (10.16)
Range	16.08–68.89	11.18–66.53	11.18–68.89
Index of Life Quality (ILQ) at 6 months, mean (SD)	44.36 (7.30)	48.13 (7.55)	47.38 (7.65)
Range	27.86–59.01	25.70–60	25.70–60
Individual psychotherapy	78 (64.5%)	304 (62.4%)	382 (62.8%)
Number of individual interviews (1–6), mean (SD)	1.80 (1.20)	1.67 (0.97)	1.70 (1.02)

Note: NYHA: New York Heart Association Classification; PCS: physical component summary; MCS: mental component summary.

**Table 2:** Results of the repeated measures mixed models on depression and anxiety from admission to discharge.

Outcome: Depression							
Fixed Effects	Beta	se	95%CI	Std. Beta	Std. se	Std. 95%CI	*p*
(Intercept)	−7.30 ***	1.13	−9.51–−5.09	0.31	0.07	0.17–0.45	<0.001 ***
Time [T1 vs. T0]	−0.78 ***	0.16	−1.09–−0.48	−0.19	0.04	−0.26–−0.11	<0.001 ***
Sex [M vs. F]	−1.15 ***	0.32	−1.78–−0.52	−0.28	0.08	−0.43–−0.13	<0.001 ***
Age	0.08 ***	0.01	0.06–0.11	0.18	0.03	0.12–0.24	<0.001 ***
Anxiety	0.24 ***	0.01	0.22–0.27	0.52	0.03	0.47–0.58	<0.001 ***
Stress	0.68 *	0.33	0.03–1.32	0.06	0.03	0.00–0.12	0.040 *
**Random Effects**							
σ^2^	4.7		ICC			0.49	
τ_00 v1cod_	4.51		N_v1cod_			451	
902 Observations; Marginal R^2^/Conditional R^2^ = 0.444/0.716							
**Outcome: Anxiety**							
**Fixed Effects**	**Beta**	**se**	**95%CI**	**Std. Beta**	**Std. se**	**Std. 95%CI**	***p***
(Intercept)	33.34 ***	2.23	28.95–37.72	0.22	0.07	0.08–0.36	<0.001 ***
Time [T1 vs. T0]	−2.22 ***	0.36	−2.92–−1.52	−0.25	0.04	−0.33–−0.17	<0.001 ***
Sex [M vs. F]	−1.09	0.69	−2.46–0.27	−0.12	0.08	−0.27–0.03	0.115
Age	−0.01	0.03	−0.07–0.05	−0.01	0.03	−0.07–0.05	0.727
Depression	1.25 ***	0.06	1.13–1.37	0.58	0.03	0.53–0.64	<0.001 ***
Stress	1.68 *	0.71	0.29–3.07	0.07	0.03	0.01–0.13	0.018 *
**Random Effects**							
σ^2^	25.69		ICC			0.42	
τ_00 v1cod_	18.77		N_v1cod_			451	
902 Observations; Marginal R^2^/Conditional R^2^ = 0.428/0.670							

Note: se = standard error; 95% CI = confidence interval at 95%; std = standardized; *p* = *p*-value; M = male; F = female; EF = ejection fraction; σ^2^ = residual variance, individual-level variability; τ_00 v1cod_: intercept variance for the grouping variable, _v1cod_; ICC = Intraclass correlation coefficient; N_v1cod_ = number of groups defined by _v1cod_; * *p* < 0.05, *** *p* < 0.001.

**Table 3:** Results of the repeated measures mixed models on physical health and mental health during follow-up.

Outcome: Physical Health Summary by SF-36							
**Fixed Effects**	**Beta**	**se**	**95%CI**	Std. Beta	Std. se	Std. 95%CI	*p*
(Intercept)	40.88 ***	3.35	34.30–47.46	−0.98	0.19	−1.35–−0.61	<0.001 ***
time [T3 vs. T2]	3.77 ***	0.54	2.71–4.84	0.42	0.06	0.30–0.54	<0.001 ***
Sex [M vs. F]	3.22 ***	0.82	1.60–4.83	0.36	0.09	0.18–0.54	<0.001 ***
Age	−0.14 ***	0.04	−0.22–−0.07	−0.15	0.04	−0.22–−0.07	<0.001 ***
Stress	−0.29 ***	0.08	−0.45–−0.13	−0.14	0.04	−0.21–−0.06	<0.001 ***
MCS	0.14 ***	0.03	0.08–0.20	0.16	0.04	0.09–0.23	<0.001 ***
EF [>35%]	3.56 *	1.5	0.61–6.50	0.4	0.17	0.07–0.73	0.018 *
**Random Effects**							
σ^2^	37.04		ICC			0.42	
τ_00 v1cod_	26.62		N_v1cod_			576	
800 Observations; Marginal R^2^/Conditional R^2^ = 0.200/0.535							
**Outcome: Mental Health Summary by SF-36**							
**Fixed Effects**	**Beta**	**se**	**95%CI**	**Std. Beta**	**Std. se**	**Std. 95%CI**	***p***
(Intercept)	37.30 ***	4.03	29.39–45.21	−0.06	0.19	−0.44–0.33	<0.001 ***
time [T3 vs. T2]	−1.06	0.62	−2.27–0.16	−0.1	0.06	−0.22–0.02	0.088
Sex [M vs. F]	0.09	0.97	−1.81–2.00	0.01	0.09	−0.18–0.20	0.922
Age	0.11 *	0.04	0.02–0.19	0.1	0.04	0.02–0.17	0.011 *
Stress	−0.81 ***	0.09	−0.99–−0.64	−0.33	0.04	−0.40–−0.26	<0.001 ***
PCS	0.18 ***	0.04	0.10–0.26	0.16	0.03	0.09–0.23	<0.001 ***
EF [>35%]	1.41	1.76	−2.05–4.86	0.14	0.17	−0.20–0.48	0.424
**Random Effects**							
σ^2^	44.06		ICC			0.48	
τ_00 v1cod_	41.17		N_v1cod_			576	
800 Observations; Marginal R^2^/Conditional R^2^ = 0.149/0.560							
**Outcome: Index of Life Quality by SF-36**							
**Fixed Effects**	**beta**	**se**	**95%CI**	**std. Beta**	**std. se**	**std. 95%CI**	***p***
(Intercept)	69.00 ***	7.15	54.96–83.04	−0.75	0.22	−1.19–−0.31	<0.001 ***
time [T3 vs. T2]	3.91 ***	1.02	1.91–5.92	0.25	0.06	0.12–0.37	<0.001 ***
Sex [M vs. F]	3.16	1.73	−0.23–6.55	0.2	0.11	−0.01–0.41	0.067
Age	−0.09	0.08	−0.25–0.06	−0.05	0.04	−0.14–0.03	0.231
Anxiety	−0.31 ***	0.09	−0.48–−0.14	−0.17	0.05	−0.26–−0.08	<0.001 ***
Depression	−0.88 ***	0.19	−1.26–−0.51	−0.22	0.05	−0.31–−0.13	<0.001 ***
EF [>35%]	6.81 *	3.23	0.47–13.14	0.43	0.2	0.03–0.83	0.035 *
Stress	−1.78	1.72	−5.15–1.59	−0.04	0.04	−0.13–0.04	0.301
**Random Effects**							
σ^2^	85.89		ICC			0.56	
z	108.06		N_v1cod_			416	
581 Observations; Marginal R^2^/Conditional R^2^: 0.204/0.647							

Note: se = standard error; 95% CI = confidence interval at 95%; std = standardized; *p* = *p*-value; M = male; F = female; EF = Ejection Fraction; PCS: physical component summary; MCS: mental component summary; σ^2^ = residual variance, individual-level variability; τ_00 v1cod_: intercept variance for the grouping variable, _v1cod_; ICC = intraclass correlation coefficient; N_v1cod_ = number of groups defined by _v1cod_; * *p* < 0.05, *** *p* < 0.001.

## Data Availability

Data Accessibility Restrictions apply to the availability of these data to ensure the privacy of the participants. The data can be requested from the corresponding Author.
